# Musculoskeletal infections through direct inoculation

**DOI:** 10.1007/s00256-024-04591-w

**Published:** 2024-01-30

**Authors:** Nuran Sabir, Zehra Akkaya

**Affiliations:** 1 Department of Radiology, Faculty of Medicine, Pamukkale University, Kinikli Kampusu, 20100 Denizli, Turkey; 2 Department of Radiology, Faculty of Medicine, İbni Sina Hospital, Ankara University, Ankara, Turkey

**Keywords:** Musculoskeletal infection, Computed tomography, Magnetic resonance

## Abstract

Musculoskeletal infections consist of different clinical conditions that are commonly encountered in daily clinical settings. As clinical findings and even laboratory tests cannot always be specific, imaging plays a crucial role in the diagnosis and treatment of these cases. Musculoskeletal infections most commonly occur secondary to direct inoculation into the skin involuntarily affected by trauma, microorganism, foreign bodies, or in diabetic ulcers; direct infections can also occur from voluntary causes due to surgery, vaccinations, or other iatrogenic procedures. Hematogenous spread of infection from a remote focus can also be a cause for musculoskeletal infections. Risk factors for soft tissue and bone infections include immunosuppression, old age, corticosteroid use, systemic illnesses, malnutrition, obesity, and burns. Most literature discusses musculoskeletal infections according to the diagnostic tools or forms of infection seen in different soft tissue anatomical planes or bones. This review article aims to evaluate musculoskeletal infections that occur due to direct inoculation to the musculoskeletal tissues, by focusing on the traumatic mechanism with emphasis on the radiological findings.

## Introduction

Musculoskeletal infections include a wide range of clinical conditions with highly significant consequences. Infection is the leading cause of fracture non-union, total joint replacement failure, and below-knee amputations [1]. The routes of introduction of infectious agents in the musculoskeletal system include hematogenous spread, direct implantation of the infectious agent, contiguous infection from infected primary sites, or in the postoperative setting which may result from either one or a combination of the first three routes [2–5]. As opposed to the pediatric population for whom the most common route of infection is hematogenous spread, in adults, direct inoculation or contiguous spread which usually involves loss of skin or mucosal integrity constitutes the most common routes for infection [3–8]. Microorganisms may be introduced into the tissues via traumatic injuries (foreign body (FB) traumas, bite/scratch or puncture wounds, irradiation, burns, open fractures, and soft tissue lacerations), biomechanic disturbances usually in relation to metabolic disorders (decubitus, vascular, or diabetic ulcers), or following iatrogenic procedures (biopsies, injections, vascular or other interventions, surgical procedures requiring orthopedic hardware) [2, 4, 6, 9–11]. Risk factors include immunosuppression, substance abuse, extremes of age, malnutrition, and obesity [2]. History may not always be remarkable and only less than one third of tissue cultures yield conclusive results for the causative agent [9, 12, 13]. Thus, imaging plays vital a role in diagnosis, assessment of the extent of involvement, guiding diagnostic and therapeutic interventions, treatment planning, and follow-up of musculoskeletal infections [2, 4, 7].

The scope of this review is to cover the role of imaging in musculoskeletal infections through direct inoculation with emphasis on common clinical scenarios in the adult population.

## Background

In the USA, annual numbers for traumatic wounds reach up to 11 million and 2–50% of open fractures are complicated by osteomyelitis [1, 14]. Moreover, there is a rise in the trend for the incidence of osteomyelitis, reaching up to about 25 per 100,000 person-years during 2000–2009 and joint infections, in parallel to increasing prevalence of diabetes in population as well as increasing use of orthopedic hardware in the reconstruction of damaged bones and joints [5, 15–18]. The rising rates of musculoskeletal infections underscore the significance of prompt diagnosis and accurately assessing the extent of disease for effective management. Consequently, the role of imaging in musculoskeletal infections, particularly with advanced techniques, is growing rapidly.

Musculoskeletal infections result from heterogeneous clinical scenarios; thus, the microorganisms vary depending on the initial source of inoculation [9]. Regardless of their route, most musculoskeletal infections are bacterial in origin [1, 3, 4, 19]. Once introduced in the sterile environment by breaking the intact skin or mucosal barrier, microorganisms may involve skin, subcutaneous soft tissues, fascia, tendon sheaths, and muscles [6]. If the infection propagates beyond soft tissues or the initial inoculation involves joints, periosteum, or bone, septic arthritis or osteomyelitis may ensue [2–8, 10, 11, 20].

Osteomyelitis is classified according to its time course (acute to chronic) or based on the route of infection (hematogenous, implant-related, or direct extension either by inoculation or contiguous spread) [4, 6, 8, 11, 20, 21]. Diabetes-related bone and soft tissue infections and implant infections pose additional diagnostic and therapeutic challenges.

**Fig. 1:**
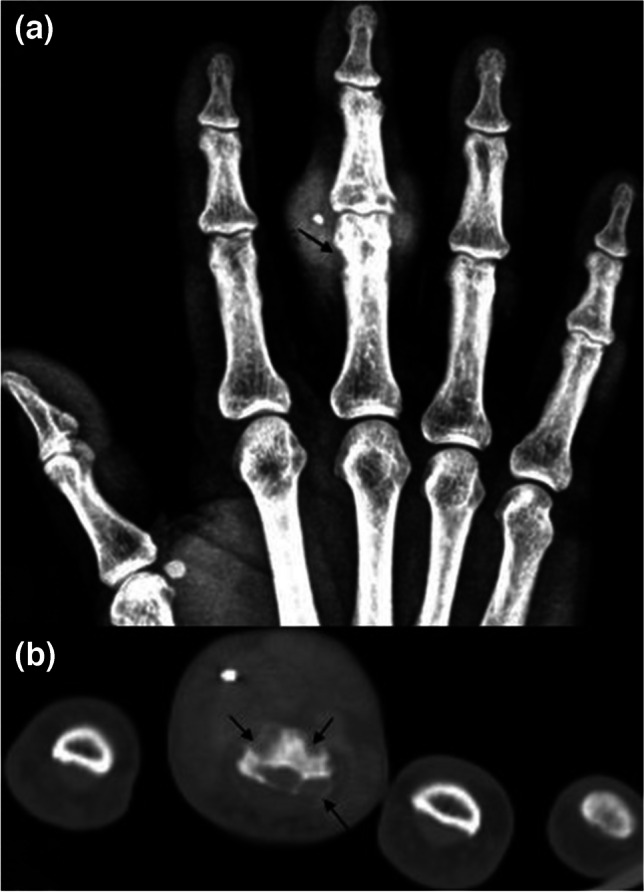
The 72-year-old man presented with prolonged pain for 2 years after hitting his hand on a solid object. A soft tissue mass-like swelling of proximal interphalangeal joint of the 3rd finger is noted. Anteroposterior radiograph (**a**) and axial bone window CT (**b**) images displayed erosions (arrows) on proximal phalanx along with a metallic foreign body impacted in the soft tissues. The large soft tissue swelling, representing foreign body granuloma, could mimic a tumor. MRI was not performed in this patient due to the potentially ferromagnetic properties of the foreign body within the soft tissue. A needle tip fragment was removed surgically

**Fig. 2:**
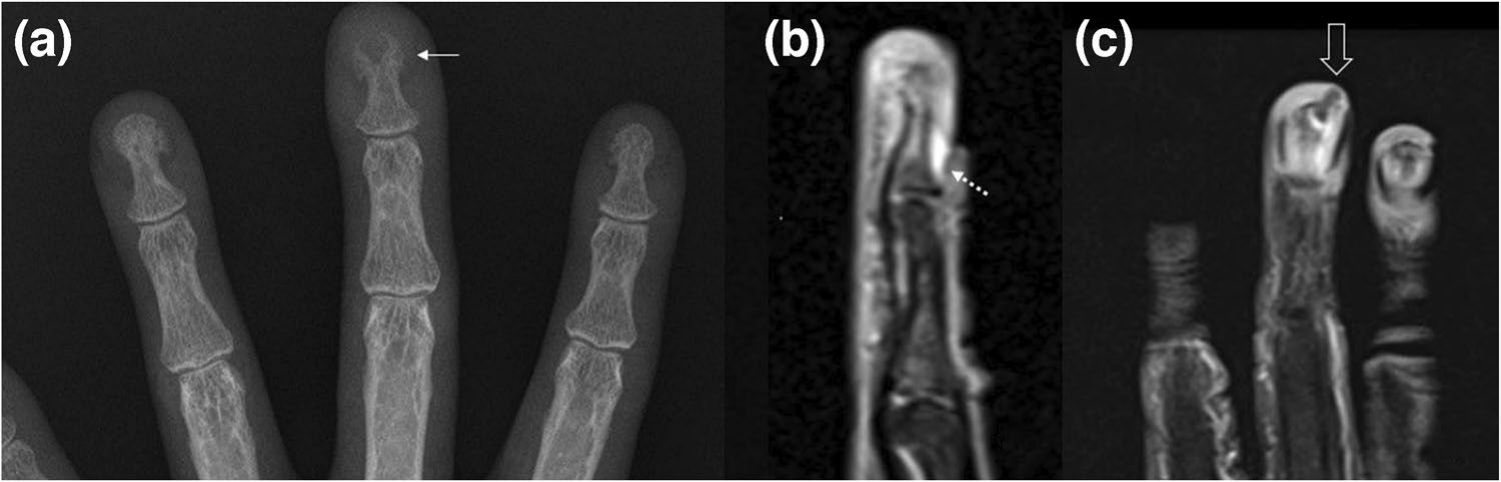
Anteroposterior radiograph (**a**) and sagittal fat-suppressed proton density-weighted (fs-PDW) image (**b**) of a 52-year-old woman with paronychia and associated osteomyelitis of the distal phalanx are shown. Note the lytic bone lesion at the tuft of 3rd distal phalanx (arrow in a) and small fluid collection in the fs-PDW image (dashed arrow) underneath the skin fold. (**c**) Coronal postcontrast fs-T1W image depicts contrast enhancement along with a micrometallic artifact (open-arrow) possibly from a previous penetrating metallic foreign body trauma, although the patient could not recall a specific history

**Fig. 3:**
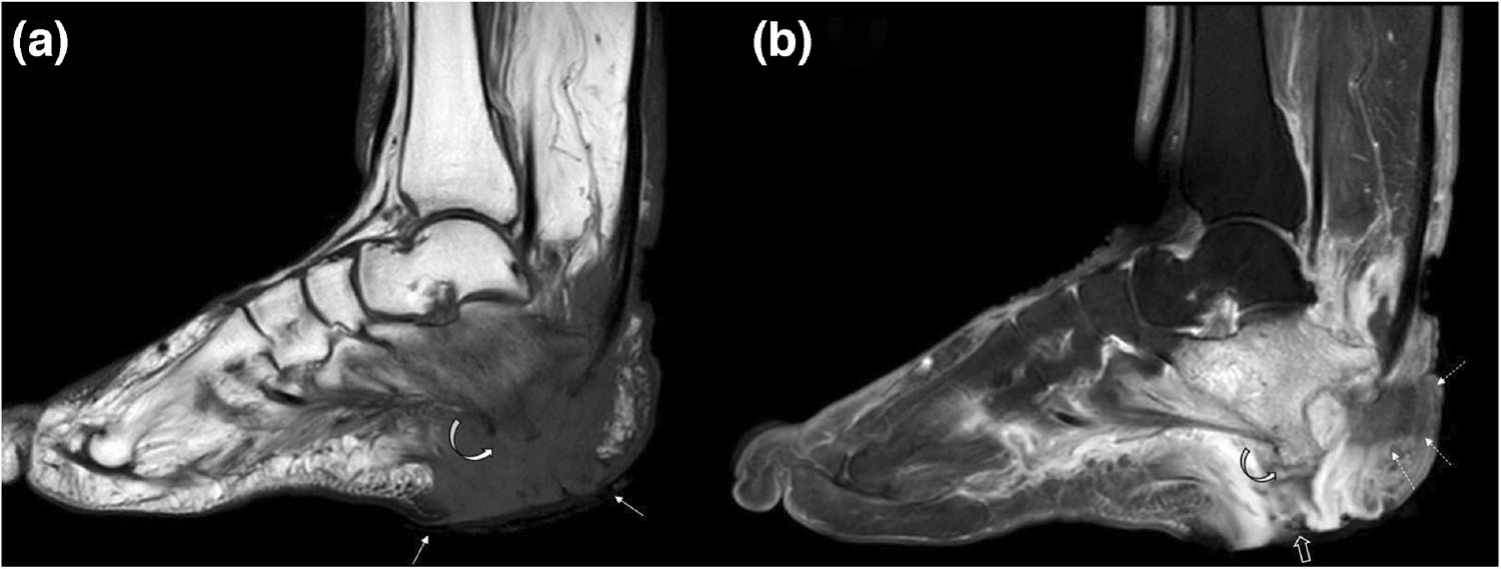
Sagittal T1W (**a**) and fat-suppressed postcontrast T1W (**b**) images of a 63-year-old diabetic woman with marked swelling and deep ulceration in the heal of the left foot are presented. Extensive pathological bone marrow and soft tissue signal changes involving the calcaneus and heel pad soft tissues are seen. The calcaneal bone marrow changes, representing osteomyelitis, are most prominent at the posterior-inferior aspect of the bone adjacent to the large ulcer (arrows) that extends to the bone through a soft tissue sinus tract (open arrow). The contour conspicuity of calcaneus at the posteroinferior aspect is more prominent (curved arrow) on postcontrast image which represents “ghost sign.” Note the small area of devitalized soft tissues (dashed arrows) near the Achilles tendon insertion

**Fig. 4:**
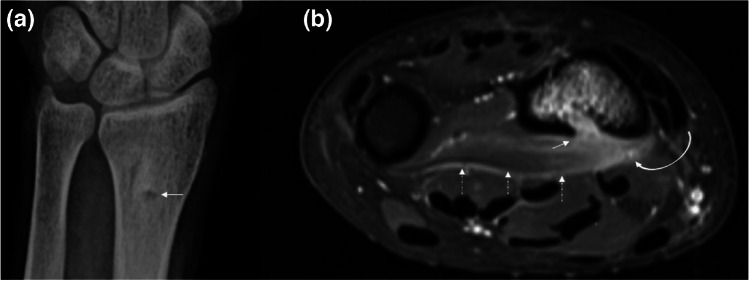
Images from a 30-year-old man, presenting with prolonged pain in the left forearm, following a dog bite 3 months ago are presented. Initial wound was locally treated for superficial lacerations in an outpatient clinic. Anteroposterior radiograph (**a**) of the wrist shows a lytic focus (arrow) with mild peripheral sclerosis on distal radius. Axial postcontrast fat-suppressed T1W (**b**) image confirms the single-sided cortical defect (arrows in a and b) that involves the volar aspect of distal radius with accompanying bone marrow edema and enhancement, compatible with acute osteomyelitis. Note the associated volar-sided soft tissue enhancement in the pronator muscle (curved arrow), deep fascia (dashed arrows) compatible with pyomyositis and fasciitis

**Fig. 5:**
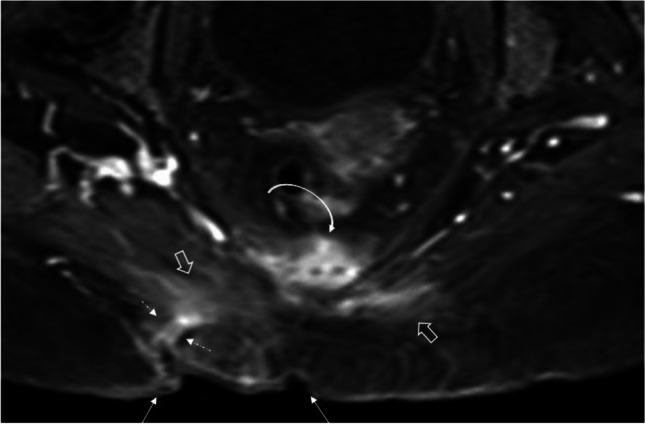
Fat-suppressed postcontrast T1W MR image shows sacral decubitus (arrows) ulcer in a 61-year-old diabetic patient. Note the irregularity of skin and the “tram-track” appearance (dashed arrows) of the sinus tract that opens to the ulcer base. Enhancement of the adjacent gluteus maximus muscles (open arrows) and sacral vertebra (curved arrow) indicate pyomyositis and osteomyelitis, respectively. Tissue cultures revealed polymicrobial etiology of *Acinetobacter baumannii* and *P. aeruginosa*

**Fig. 6:**
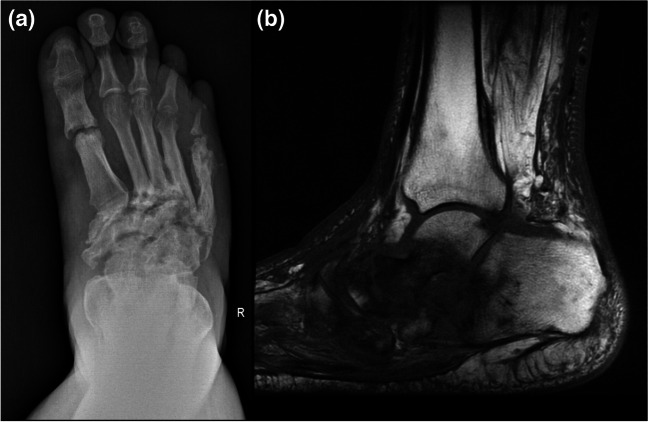
Anteroposterior radiograph of the foot (**a**) shows destruction and deformity of the tarsal and metatarsal bones with sclerosis, joint space narrowing, cortical irregularities, and fragmentation, Lisfranc injury involving the second to fifth tarsometatarsal joints with lateral dislocation in a patient with neuropathic osteoarthropathy. Sagittal T1W MR image (**b**) reveals a collapse in the longitudinal arch of the foot with a generalized bone marrow edema throughout the tarsal and metatarsal bones in the midfoot

**Fig. 7:**
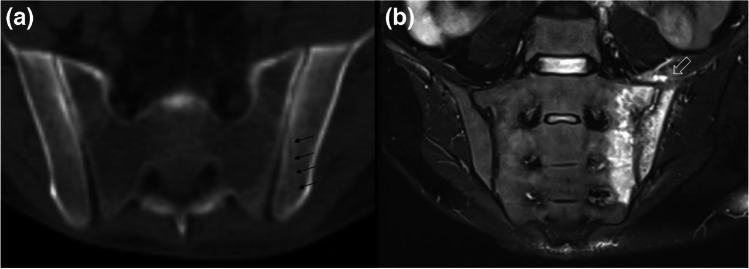
Axial CT image (**a**) depicting the needle track (black arrows) in a patient who underwent bone marrow biopsy and later developed postbiopsy septic sacroiliitis. Coronal STIR (**b**) image shows unilateral, extensive bone marrow edema on both sacral and iliac sides of the left sacroiliac joint, with joint effusion causing capsular distension (open arrow) and associated inflammatory signal changes in the left iliacus muscle. Pronounced periarticular inflammatory soft tissue changes, extensive edema, and asymmetric unilateral involvement are in favor of septic sacroiliitis and the needle track in the CT image acquired prior to start of clinical symptoms suggests iatrogenic seeding of infection in this patient. Joint fluid cultures revealed *S. aureus* as the etiologic microorganism

**Fig. 8:**
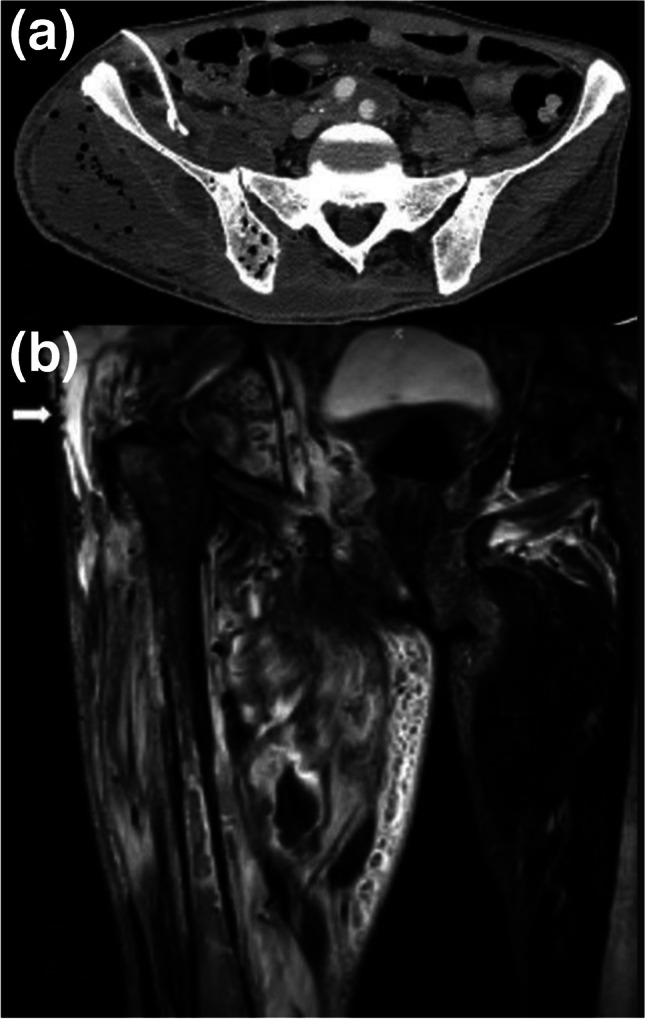
Axial CT (**a**) and coronal STIR (**b**) MR images of a 56-year-old man 3 months postangiography, performed via right femoral artery by Seldinger technique, are shown. Note the marked enlargement of the right gluteal and thigh muscles with low-density areas and air bubbles, compatible pyomyositis with abscesses and necrotizing fasciitis. The intraosseous air in the right iliac bone can be appreciated on the CT image as well as the drainage catheter in the right iliacus muscle abscess. Coronal STIR image (b) depicts that the extensive high-signal areas within superficial and deep soft tissue compartments, stranding within subcutaneous fat with a fluid collection in the right gluteal region (arrow), confirm pyomyositis, abscess, and necrotizing fasciitis and cellulitis. Multiple air sacs appear as signal-void areas within the medial thigh muscles on the MR image (b)

**Fig. 9:**
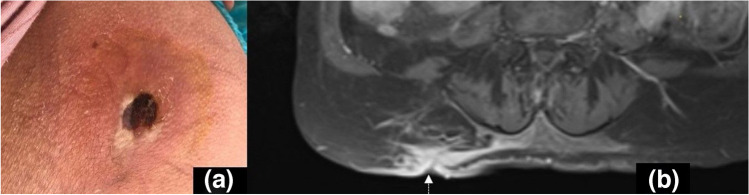
Photograph (**a**) of a right gluteal ulcer in a 62-year-old patient with acute myeloid leukemia at bone marrow aspiration biopsy site. Corresponding axial fat-suppressed postcontrast T1W image (**b**) depicts the thickening of the extent of involvement of right gluteal subcutaneous fat and superficial soft tissues. The well-demarcated and enhancing ulcer cavity (dashed arrow) and surrounding reticular enhancement representing cellulitis involve a larger area than the size of the skin ulcer

**Fig. 10:**
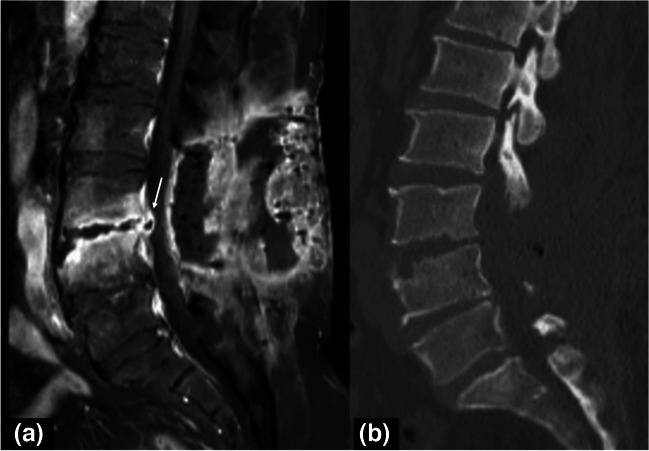
Sagittal fat-suppressed postcontrast T1W MR (**a**) and corresponding sagittal reformatted CT image in bone window (**b**) reveal findings of spondylodiscitis in a 65-year-old women with a recent history of operation on herniated L3-4 intervertebral disk who presented with back pain and discharge at the operation site. Note the large soft tissue abscess with peripheral rim enhancement extending all the way between skin to posterior epidural space at the level of L3-L4 vertebral bodies. There is also an anterior epidural abscess (arrow) and marked enhancement in L3-L4 endplates. CT image (b) better demonstrates end-plate irregularities and bony erosions

**Fig. 11:**
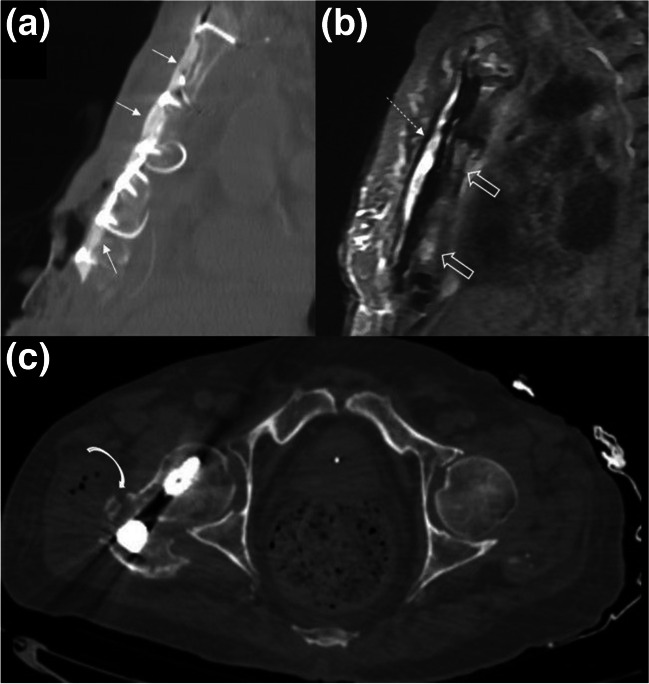
Sagittal reformatted CT (**a**) and multi-acquisition with variable-resonance image combination (MAVRIC) proton density fluid MR image (GE Healthcare) (**b**) of a patient with sternal osteomyelitis and presternal soft tissue infection, 3 months after cardiac surgery, are shown. The soft tissue defect at the lower quadrant of sternal surface is appreciated on CT image. Antibiotic-eluding bone cement and metallic wires were used to reconstruct the sternal defect which can be appreciated on CT image (arrows). Using a metal artifact reduction techniques on MRI, collection overlying the bone cement (dashed arrows) and edema-like signal changes in the presternal soft tissues can be appreciated. Note the relatively insignificant susceptibility artifacts due to the metallic wires on this sequence. Underlying corpus sterni shows high signal intensity areas (open arrows) which suggest sternal osteomyelitis. Axial CT image (**c**) in bone window in another patient with infected right hip prosthesis demonstrates periarticular collection with air bubbles and bone fragmentation at the anterior aspect of greater trochanter (curved arrow)

**Fig. 12:**
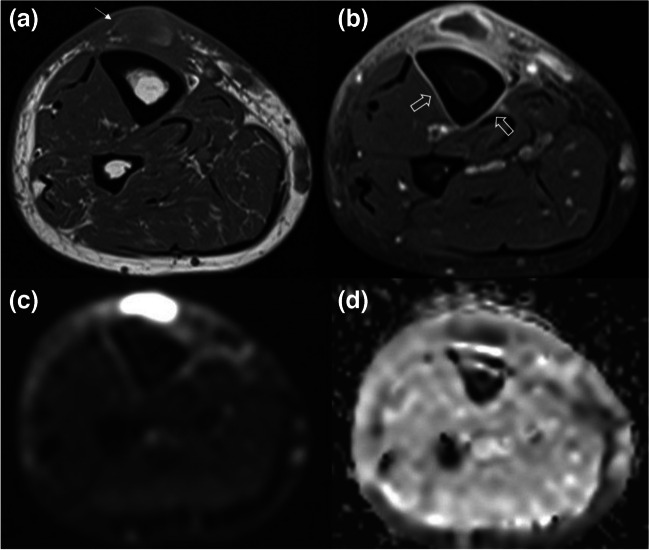
Images of a 45-year-old man who presented to orthopedic clinic with a soft tissue swelling and erythema on the right pretibial area 1 month after a fall which resulted in laceration of the skin at the same location are presented. Initial laceration had been treated with sutures elsewhere. Axial precontrast (**a**) and postcontrast fat-suppressed T1W (**b**), representative diffusion-weighted image (**c**), and apparent diffusion coefficient (ADC) map (**d**) are shown. The abscess has a hyperintense wall on precontrast T1W image (arrow) representing the “penumbra” sign. Rim enhancement on postcontrast image and diffusion restriction in the abscess cavity further support the nature of this collection. Note that the adjacent tibial periosteum also enhances, representing periosteal reaction (open arrows in b)

**Fig. 13:**
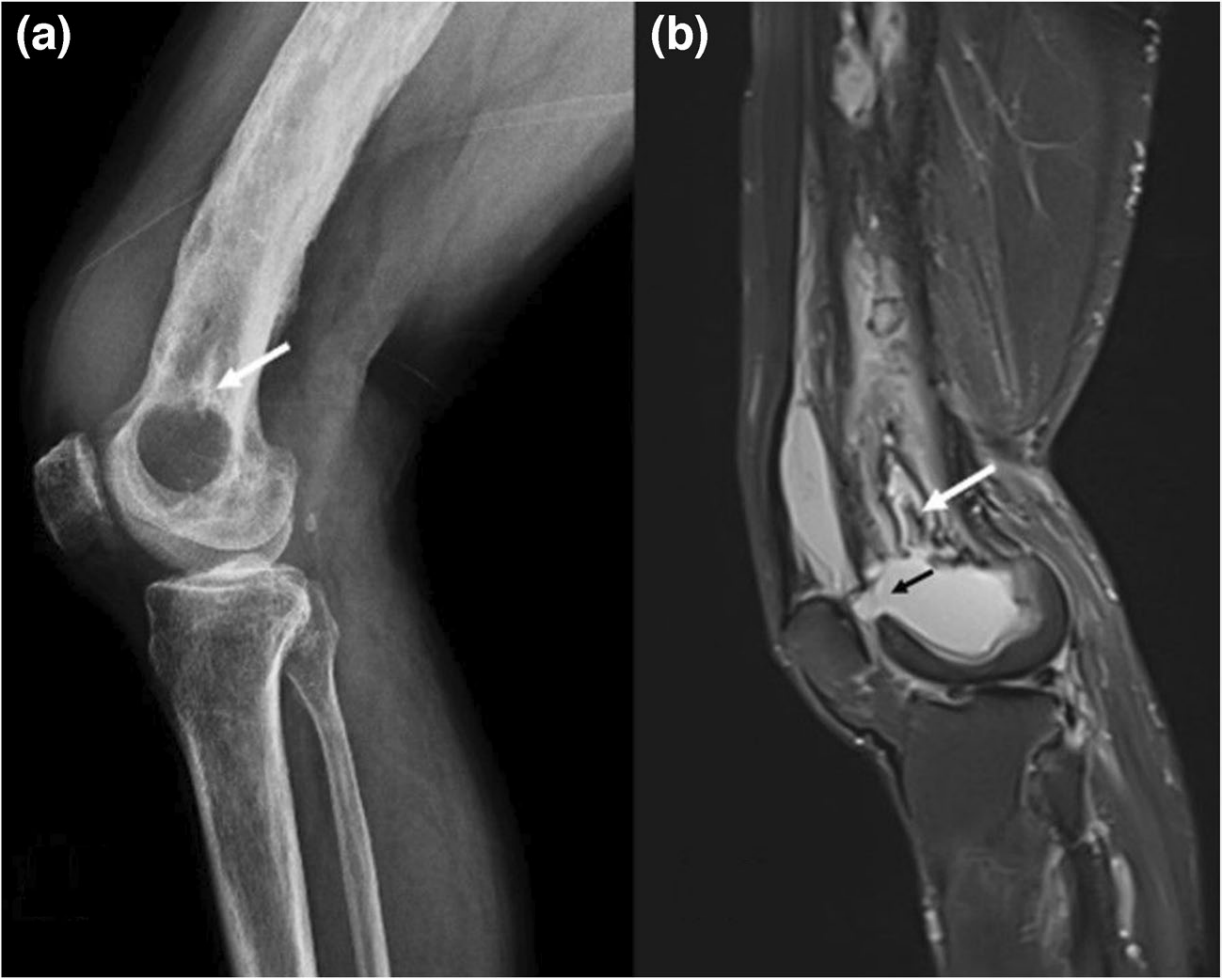
Lateral radiograph (**a**) and sagittal fat suppressed T2W MR image (**b**) of distal femur and knee joint of a 70-year-old man who suffered a trauma to the right femur 20 years ago are shown. Compatible with chronic osteomyelitis, distal femur shows sclerosis, cortical thickening and irregularity, and slight bowing deformity with a large lucent lesion in the distal metaepiphysis. The linear sclerotic lesion (white arrow) represents a sequestrum that lies immediately superior to the fluid-filled space which corresponds to the lucent lesion on the radiograph. This cystic lesion showed peripheral rim enhancement (not shown here) and represents Brodie’s abscess. Note the small defect on its anterior wall representing a cloaca (black arrow), through which the abscess cavity communicates with the suprapatellar bursa of the knee. The patient also had swelling, pain, and erythema of the knee joint compatible with septic arthritis as a complication

**Fig. 14:**
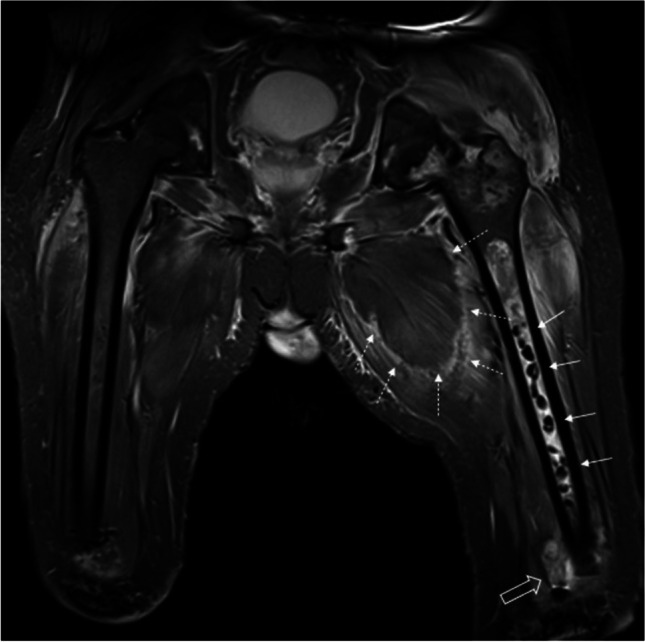
Coronal STIR image depicting left femoral osteomyelitis 3 months after bilateral distal femoral amputations in a 25-year-old male survivor from a crush injury. Antibiotic-eluding beads, which may mimic sequestered bone fragments (arrows), were placed into the medullary cavity of left femur after removal of an infected femoral implant which was initially placed in early posttraumatic period (not shown here). Patient then required amputation for further complications in the left lower extremity. Note the small soft tissue collection (open arrow) near stump on the left side. The extensive abnormal signal intensity in the proximal thigh and gluteal muscles reflect areas of myonecrosis (dashed arrows), best demonstrated on the adductor group on this coronal STIR image. Postcontrast images could not be acquired as the patient developed acute compartment syndrome and consequent renal failure due to rhabdomyolysis. The left femoral neck bone marrow signal changes most likely reflect osteonecrosis in this patient. Myonecrosis may show peripheral rim enhancement on postcontrast images and should not be mistaken for soft tissue abscess. Lack of central T2 hyperintensity could help to distinguish myonecrosis from abscess

In 2021, in an effort to standardize the terminology, an expert panel appointed by the Society of Skeletal Radiology Practice Guidelines and Technical Standards Committee published a White Paper on the recommended and discouraged magnetic resonance imaging (MRI) nomenclature on elementary lesions of peripheral musculoskeletal infections (Figs. 1, 2, 3, 4, 5, 6, 7, 8, 9, 10, 11, 12, 13, and 14) [6]. The terms emphasized in this review include “edema”/ “cellulitis,” “ulcer,” “sinus tract,” “soft tissue abscess,” “devitalized soft tissues,” and “necrotizing fasciitis” for the soft tissue lesions; “septic arthritis,” “synovitis,” “septic/infectious tenosynovitis/infectious paratenonitis,” and “erosions” for joints and periarticular soft tissue lesions; and “periosteal reaction,” “subperiosteal abscess,” “cloaca,” “osteomyelitis,” “Brodie’s abscess,” “devitalized bone/sequestrum,” and “involucrum” for bone lesions. Presence of certain elementary lesions (i.e., ulcers) can serve as a clue to the infection route and increase diagnostic confidence for other lesions (i.e., subtle bone marrow T2 signal changes on MRI might indicate osteomyelitis if next to an ulcer or sinus tract). Whereas other lesions such as periosteal reaction can manifest similarly irrespective of the direction of inciting infective process (centripedally from soft tissue towards cortical bone or centrifugally from medullary cavity towards cortex). Furthermore, the expert panel suggested replacement of some terms such as “phlegmon” which indicates an “ill-defined inflammatory mass-like lesion reflecting the acute or infiltrative phase of infected soft tissue, prior to liquefaction and pseudocapsule formation,” by more descriptive terminology such as “cellulitis/myositis/fasciitis without abscess.” Of note, “pyomyositis,” indicating bacterial infection of skeletal muscles either primarily or through contagious spread, was defined but not listed as a separate terminology. Moreover, specific recommendations on prosthetic joints or implant infections were not included in this review [6]. Table 1 summarizes the recommended terminology and special imaging concerns.

**Table 1:** The elementary lesions in musculoskeletal tissue infections and their recommended terminology by the Society of Skeletal Radiology Expert Committee [6]

Recommended terminology		Description	Findings and recommendations for MR imaging
Soft tissues	Edema	Excessive amount of fluid in interstitial space in localized or generalized form	Reticular areas of fluid signal intensity on all sequences and lack of enhancement in postcontrast images
	Cellulitis (Figs. 2, 3, 9, and 12)	Non-necrotizing superficial (above deep fascia) bacterial soft tissue infection	Ill-defined, reticular areas of fluid signal in superficial fascia and subcutaneous fat resembling bland edema but with enhancement in postcontrast images
	Ulcer (Figs. 3, 5, and 9)	Discontinuation of skin, epithelium, or mucous membranes	Area of focal surface discontinuity on skin or a soft tissue defect On MRI, T2-hyperintense and enhancing margins represent granulation tissue; lack of enhancement could be a sign of tissue devitalization or scar formation A useful approach for planning an MR study as well as detecting small skin lesions could be placing a marker at the site of ulcer or fistula opening
	Sinus tract (Figs. 3 and 5)	A channel extending from skin/mucosal surface to deeper region of suppurative infection	Linear structure containing fluid, granulation, or necrotic tissues between area of suppuration (in bone or soft tissues) to the skin surface “Tram-track” appearance of peripheral enhancement on postcontrast MRI Imaging in all planes is recommended
	Abscess^3^(Figs. 2 and 12)	Collection of pus in tissues due to pyogenic infection	Well-defined area of iso-hypointense area on T1W and hyperintense signal on T2W images, peripheral enhancement on postcontrast MRI Subtraction images after IV contrast administration and diffusion restriction on DWI could increase their conspicuity and diagnostic confidence “Penumbra sign” is referred to the relatively hyperintense abscess walls on precontrast T1W images The use of phrases suggesting the “drainability” of the collection in radiology reports based solely on MRI findings is discouraged In patients who cannot be imaged by MR, contrast-enhanced CT could also present the rim-enhancing fluid collections [2]
	Devitalized tissue (Fig. 3)	Covers both necrotic and ischemic soft tissues	Geographic area showing lack of enhancement or rim-enhancement and clear-cut margins between vital (enhancing) tissues on postcontrast MRI Almost entirely in the setting of diabetic or peripheral vascular disease infections Caution should be applied to identify confounding causes of disturbed tissue perfusions such as external pressure on the tissue or venolymphatic congestion, which may have similar MRI appearance
	Necrotizing fasciitis (Fig. 18)	Aggressive, potentially life-threatening bacterial infection involving superficial and deep tissue compartments	Wide availability, rapidness, and superiority in detection of soft tissue gas renders CT as the first choice of imaging modality Both CT and MRI could depict fascial fluid or gas collections, fascial thickening (≥ 3 mm), and fat infiltration. On MRI, focal and diffuse non-enhancement of fascial planes, involvement of ≥ 3 compartments in one extremity increase diagnostic confidence Most important prognostic factor for mortality is delay in diagnosis, which reaches 70–80% [68]
Skeletal tissues	Periosteal reaction	New bone formation at the periosteum in reaction to an infection	May appear as thickening, lamellated, or aggressive in pattern as in the formation of Codman’s triangle
	Subperiosteal abscess	Collection of pus in the subperiosteal space between periosteum and the cortex	On US, mixed or hypoechoic fluid collection between periosteum and cortex On CT and MRI abnormal fluid collection in the subperiosteal space with findings similar to abscesses elsewhere
	Cloaca (Fig. 13)	A defect in the cortex of the infected bone that allows the drainage of pus from bone into surrounding soft tissues	On radiographs and CT, a lucent gap or a breach in the cortex On MRI, appears as a gap in cortex of bone as low signal on T1W and high signal on T2W or fluid-sensitive sequences with enhancement on contrasted sequences Cloaca is a sign of chronic osteomyelitis Upon successful treatment it can heal by callus formation
	Involucrum	Area of thickened viable bone that forms around the necrotic infected bone	On radiographs and CT, an irregular thickening of the cortex On MRI, the thickened outer layer of an involucrum representing new healthy bone which follows cortical signal. The inner granulation layer and periosteal new bone formation may appear as a high-signal rim on T1W images
	Osteomyelitis (Figs. 2, 3, 4, 5, 13, and 14)	Infection of bone involving the medullary canal mostly due to bacterial proliferation	Acute, subacute, or chronic depending on the time scale of the infection Early radiographic findings of acute osteomyelitis may be subtle, and may take up to 3 weeks to be appreciated They include local osteopenia, trabecular destruction, lytic changes with ill-defined margins, and lamellated periosteal reaction [59] MRI is most sensitive in detecting the early changes of osteomyelitis like bone marrow edema as early as 1–2 days of starting of infection. It appears as an area low-T1 and high-T2 signal which enhances on postcontrast images If present “ghost sign” favors osteomyelitis and can help in distinguishing diabetic foot osteomyelitis from NA, or superimposed osteomyelitis on existing NA [5] T1W sequences provide good anatomical detail and enable delineation of the medulla, cortex, periosteum, and soft tissues
	Sequestrum (Fig. 13)	A separated fragment of devascularized bone which is surrounded by pus, granulation tissue, and an involucrum	On radiographs and CT sequestrum can be seen as a fragment of separated bone surrounded with a low attenuating rim of granulation tissue On MRI, devascularized fragment appears as a low-intensity structure without enhancement after IV contrast administration. The surrounding granulation tissues may enhance Sequestra are signs of chronicity
	Intraosseous/Brodie’s abscess (Fig. 13)	An intraosseous abscess related to a focus of subacute or chronic pyogenic osteomyelitis	Has a predilection to the metaphyseal ends of the tubular bones On radiographs and CT, it appears as a lytic lesion with sclerotic dens rim. Periosteal reaction and soft tissue swelling may accompany On MRI, “penumbra sign” can help in differentiating Brodies abscess from other bone lesions. It is referred to as hyperintense rim lining of the abscess walls on precontrast T1W images which strongly and rapidly enhance after contrast administration
Joints and periarticular soft tissues	Synovitis	Synovial thickening due to increase in the cellularity of the synovial membrane in the setting of infectious synovial proliferation	Suggested MR imaging for assessing synovial disease includes precontrast and postcontrast T1W images and T2W fat-saturated or STIR images in at least two different planes On MRI, synovitis seen as thickened and irregular layer of tissue, with avid enhancement on postcontrast study
	Infectious/septic tenosynovitis or paratenonitis^1^	Fluid within the tendon sheath with synovial thickening	Plain radiograph has a limited value in the diagnosis of infective tenosynovitis as it can only display soft tissue swelling and/or gas or foreign bodies as a potential cause US may show synovial hyperemia and fluid distension of the tendon sheath CT demonstrates fluid and/or gas within the tendon sheath, synovial thickening, and enhancement MR is recommended as it demonstrates fluid signal within the tendon sheath, soft tissue edema around the tendon sheath, tendon sheath thickening with contrast enhancement, and thickened tendons and/or enhancement
	Septic arthritis^2^(Fig. 7)	Destructive arthropathy caused by an intraarticular infection	Radiographs show non-specific findings like soft tissue swelling and joint effusion Absence of joint effusion on US is highly negative predictive MRI findings include joint effusion, synovial thickening, and surrounding soft tissue changes. Contrast administration may show diffuse synovitis and soft tissue abscess formation Diffuse marrow edema, especially if observed on T1W images, is suggestive of osteomyelitis CT and MRI can aid in assessment of difficult-to-access joints such as sacroiliac joint; furthermore, CT can aid in joint fluid aspiration [23]

^1^For tendons lacking a tenosynovial sheath, infection of the tendon can also be used

^2^The term “septic sacroiliitis” is considered acceptable

^3^Pyomyositis is not defined separately in the Expert Committee White Paper but is defined as primary infection of the muscle that should be considered in the differential diagnosis of soft tissue abscess

*FOV*: field of view, *T1W*: T1-weighted, *CT*: computed tomography, *MRI*: magnetic resonance imaging, *US*: ultrasound, *NA*: neuropathic osteoarthropathy

## Anatomy and pathophysiology

Acral regions (hands, feet, wrists) are commonly affected by infections secondary to FB traumas and puncture wounds (Figs. 1 and 2). Common FB that can act as an infective nidus include wood splinters, metal, glass, or plastic [4, 9, 22–24]. In the foot, FB, or puncture wounds, skin ulcerations characteristically involve the plantar aspect. Diabetes is a particularly important co-morbidity when considering foot infections (Fig. 3) [4, 10, 25–27].

With an incidence ranging from 0.6 to 14.8%, infection is the most common complication of retained FB, which constitutes about one third of all emergency department admissions [14, 28]. Because of their porous structure, organic materials (i.e., wood) are more likely to serve as infective niduses than inorganic ones (i.e., glass) [14, 29, 30]. Almost 40% of FB injuries to the hand are due to wood [14, 30, 30]. Metallic FB are commonly associated with gun-sot injuries [7]. Simple soft tissue infection in the terminal pulp (“felon”) and subcuticular abscess of the nail fold (“paronychia”) may progress to osteomyelitis and bone destruction (Fig. 2) [4].

Domestic or wild animal bite/scratch injuries are defensive injuries, thus commonly involve distal aspects of upper extremities (Fig. 4) [31]. Dog and cat bites account for up to 90% and 10% of all mammalian bite injuries, respectively. Most bite injuries consist of simple soft tissue lacerations and abrasions, leading to cellulitis but about 5% of dog bites and 20–50% of cat bites lead to significant infections. Simple bite wounds from a non-threatening pet are neglected more often by the patients which may paradoxically lead to significant deep and necrotizing tissue infections [22].

Following an injury to the hand, infection may disseminate via tendon sheaths, fascia, and lymphatics. Infectious tenosynovitis of the finger may result from a puncture wound in the flexor crease of the digits where the tenosynovium lies very near to the skin and cause severe pain, restricted full extension, and fusiform swelling of the finger when infected. In such a case, adjacent joints (typically the distal interphalangeal joint) and bone (typically middle phalanx) infections may ensue. Metacarpophalangeal joint involvement is less common with puncture wounds to the finger. In contrast, human bite-related injuries, which result in skin laceration at the dorsal aspect of metacarpophalangeal joints, following a blow to the mouth during a fistfight, most commonly result in septic arthritis of metacarpophalangeal joints [2, 4, 6].

Pressure ulcers (also known as decubitus ulcer or bedsores) typically begin with soft tissue ulcers where the integrity of skin is breached at specific sites of pressure, especially in patients on prolonged bed rest. Common anatomical locations include the pelvis, particularly near the sacrum, ischial tuberosities, trochanteric, and gluteal regions (Fig. 5). When osteomyelitis ensues, it typically involves the areas of bone, adjacent to the skin ulcerations, frequently at the innominate bone, proximal femora, and lower sacrum or calcanei [4, 6]. Osteomyelitis related to diabetic foot infections are also common in areas of biomechanical stress such as metatarsal heads, toes, or calcaneus (Fig. 3) [32]. Subsequently, unlike in infants and young children, who typically have osteomyelitis of the long bones, in adults osteomyelitis commonly involves spine, pelvis, hand, and foot bones [6].

## Common pathogenic agents in direct inoculation-related musculoskeletal infections

In contradistinction to hematogenous spread, infections due to inoculation or contiguous involvement are commonly polymicrobial [1, 8]. Most common culprits are Gram-positive microorganisms, particularly *Staphylococcus aureus*. It is the most common infectious agent in open fractures, pyomyositis, and implant/prosthesis-related infections, followed by coagulase negative staphylococci which are skin colonizers and Gram-negative bacilli [1,8,20,33 15,18,20,34]. Gram-negative bacterial infections (i.e., *Pseudomonas aeruginosa*, *Enterobacter* spp., *Proteus* spp.) are relatively uncommon, but are implicated in the setting of tissue ischemia, microvascular disease, or immunosuppression. Diabetic wound infections are typical examples of such states; thus, Gram-negative bacterial infections are more severe and harder to treat [4, 8, 18, 25, 26, 33]. Polymicrobial etiology and low rates of culture positivity may necessitate further expensive laboratory tests such as genomic sequencing-based methods for identification of causative organisms [1, 8, 18, 20, 34].

Fungal infections via direct inoculation are rarer but they have been reported as nosocomial infections associated with surgical indwelling materials, in trauma victims, intravenous drug users, as a complication of parenteral treatment, or odontogenic infections [35]. They are commonly associated with axial skeleton, head, and neck involvement [8, 35].

Table 2 summarizes the common pathogenic microorganisms that have been reported for various clinical scenarios of musculoskeletal infections by direct inoculation.

**Table 2:** Most common microorganisms associated with musculoskeletal infections via direct inoculation mechanism

Clinical scenario	Most common microorganisms		Notes
Cellulitis [6]	β-hemolytic streptococci MSSA/MRSA		*Pseudomonas aeruginosa* and other microorganisms in soil and normal flora are usually implicated Up to 80% of foot and ankle infections are polymicrobial
Diabetic foot ulcers, with or without osteomyelitis [4, 8, 25, 26, 33]	*S. aureus* *S. epidermidis* Anaerobic bacteria Gram-negative microorganisms		
Head and neck osteomyelitis [8, 35]	*Aspergillus* spp. *Candida* spp. *Pseudomonas* spp. *Actinomyces* spp.		Fungal musculoskeletal infections are more difficult to treat due to commonly delay in diagnosis, biofilm formation in tissues
Pyomyositis [6, 61]	*S. aureus* *Clostridium* spp.		Most common pathogen is *S. aureus* Myonecrosis usually involves Clostridium infections [69]
Puncture wounds with organic foreign bodies [70]	*Sporothrix schenckii*		Ubiquitously in nature, infections reported in gardeners, minors, and construction workers
Zoonotic bite and scratch infections [19, 22, 31, 45, 71]	Dog	*S. aureus* *Fusobacterium* *Pasteurella*, *Capnocytophaga*, *Bacteroides* species	Dog bites cause crush injury in the tissues leading to tissue ischemia and necrosis which may be followed by secondary infections. Cat bites are sharper and penetrative due to their smaller teeth which may lead to deep tissue infections and osteomyelitis more commonly than dog bites. However, osteomyelitis secondary to dog or human bites are expected to be more extensive, thus difficult to treat *Bartonella henselae* is the etiologic agent in cat-scratch disease
	Humans	*S. aureus*, *Fusobacterium*, *Streptococcus*, *Peptostreptococcus*, *Eikenella* species	
	Cat	*Pasteurella*, *Capnocytophaga*, *Bacteroides* species, *Bartonella* henselae	
	Fish Crustaceans *Echinodermata*	Atypical mycobacterial infections	*Mycobacterium marinatum* infection can arise following sea urchin spine punctures Granulomatous skin, tenosynovium, joint, or bone infections Joint infections resemble tuberculous septic arthritis with granulomatous synovitis and peripheral bone erosions where joint space is preserved [31, 72]
	Reptiles	*Salmonella*	
	Rats and invasive rodents	*Streptobacillus moniliformis* *Spirillium minus* *Leptospirosis*	Highly infective Children can be seriously injured They can cause rabies, tetanus, or sepsis due to systemic infections
Open fractures [1, 8]	*S. aureus* MSSA/MRSA *Acinetobacter baumannii* *Enterobacter* spp. *Enterococcus* spp. Multi-drug-resistant Gram negatives Gram negatives (*Pseudomonas aeruginosa*, *Enterobacter cloacae*, *E. coli*), Gram positives (*Bacillus* and *Enterococcus* spp.), anaerobes (*Clostridium* spp.), non-tuberculous mycobacteria *Acinetobacter baumannii*		Usually polymicrobial High-grade open fractures with wide-spread soft tissue injury may be further complicated by nosocomial infections by MSSA or MRSA and multi-drug-resistant Gram negatives With gross environmental contamination Combat-injury related osteomyelitis further carries risk of infection by particularly drug-resistant agents such as *Acinetobacter baumannii*
Postintervention soft tissue infections [41, 67, 67]	Beta-hemolytic *Streptococci* Skin flora elements		Antibiotic prophylaxis might be considered prior to intervention SIR guidelines recommend routine use of antibiotics prior to vertebroplasties [40]
Pressure ulcers [73]	*S. aureus* *Proteus mirabilis* *Pseudomonas aeruginosa* *Enterococcus faecalis*		Usually polymicrobial
Septic arthritis [23]	*S. aureus* *S. epidermidis*		Direct inoculation/contagious spread of bacteria into a joint is less common than hematogenous spread Common scenarios include arthrotomy, open fracture, arthrocentesis, and intraarticular injections
Implant- or prosthesis-related bone and joint infections [1, 8, 18, 20, 69, 74, 75]	*S. aureus* *Enteroccocus* spp. *Pseudomonas aeruginosa* *Escherichia coli*		Early postoperative infections > 50% of prosthetic joint infections 20% of prosthetic joint infections are polymicrobial *P. aeruginosa* is notorious for its biofilm formation
	Coagulase-negative staphylococci Streptococci Enterococci Anaerobes		Late implant-related infections are typically caused by coagulate-negative staphylococci, anaerobes, and less virulent microorganisms
	Candida Brucella Mycobacterial infections		Rare

*MRSA*: methicillin-resistant *Staphylococcus aureus*, *MSSA*: methicillin-sensitive *Staphylococcus aureus*, *spp*: species, *S. aureus*: *Staphylococcus aureus*

## Diabetic foot infections

Diabetic patients are particularly prone to foot infections resulting from direct inoculation as contiguous spread from the adjacent skin ulcers which are commonly associated with peripheral vascular disease, peripheral neuropathy, and cumulative trauma [6, 26, 27, 33]. Differentiating diabetic foot infections (Fig. 3) from neuropathic osteoarthropathy (NA) (Fig. 6) is often difficult, but early diagnosis of infection is important for appropriate management [32, 36, 37]. In up to 25% of all diabetic foot infections soft tissue necrosis and devitalization can be seen (Fig. 3) [6]. Osteomyelitis in diabetic foot infections results with extension of soft tissue infection into the bones; thus, presence of infectious soft tissue findings can improve diagnostic confidence for osteomyelitis (Fig. 3). Tracing the ulcer or sinus tract to the underlying bone and looking for the presence of marrow edema, as evidenced by low signal intensity on T1W and corresponding high signal intensity on T2W images, is the best way to diagnose osteomyelitis. “Ghost sign” suggesting osteomyelitis, which may or may not be superimposed on NA, represents bones where they “disappear,” meaning they lose their contour conspicuity on T1W images, then “reappear” on postcontrast or T2W images [5, 6, 38]. It can also be noted on apparent diffusion coefficient (ADC) maps of diffusion-weighted imaging (DWI) [39].

Intertarsal, tarsometatarsal, and metatarsophalangeal joints of the forefoot are commonly involved sites in NA (Fig. 6), with sparing of interphalangeal joints. In contrast, osteomyelitis associated with diabetic foot ulcers commonly involves calcaneus, metatarsal heads, or toes (Fig. 3) [32, 37].

## Iatrogenic and postoperative infections

### Iatrogenic infectious complications in musculoskeletal system

Musculoskeletal sites are sterile; thus, guidelines recommend the utmost care when performing interventional procedures involving these tissues [40]. Rate of infections following musculoskeletal interventions is < 1% [41]. Inadequate skin preparation can lead to the transmission of skin flora bacteria into deeper tissues during percutaneous interventions (Figs. 7, 8, and 9) [41]. Potential sources of infection can be as basic as surgical gloves, ultrasound gel, probes, and probe covers or could be related to equipment and materials that are used to access or treat the desired tissues such as injectables, biopsy needles, catheters, and orthopedic hardware. Society of Interventional Radiology (SIR) guidelines do not recommend routine prophylactic antibiotic use except vertebroplasties [40]. Extra precautions should be taken if interventions involve infected skin or musculoskeletal tissues and avoided whenever possible [40–42].

### Postoperative spine infections

Postoperative infections are common and can occur at different time intervals after the surgery. Some are related to instrumentation, others due to procedure and/or the approach. Risk factors for these infections following surgery may include surgical length, use of drains, retained fragments following trauma, immunosuppression, and previous irradiation [43].

Radiographs are insensitive in the early changes of spondylodiscitis. CT can show soft tissue swelling, intervertebral disk enhancement, and epidural collections. MRI is the modality of choice to diagnose spondylodiscitis because it displays early changes in the vertebral bodies, intervertebral disk, paravertebral area, and epidural region (Fig. 10). Early inflammation of the disk is manifested by increase in its T2 signal intensity owing to increase its water contents and avid enhancement in postcontrast images.

### Prosthetic joint and orthopedic hardware related peripheral skeletal infections

Incidence of infections with implant surgery ranges between 0.5 and 2.4% but it reaches up to 20% in revision procedures [18, 23]. Bacteria may be introduced directly into the joint during the surgery or later by hematogenous spread (Fig. 11). In fact, the route of contamination is unknown in most cases but early infections within the first 3 months highly likely represent inoculation during surgery [16, 18, 23]. Risk factors for prosthetic joint infections (PJI) include male sex, smoking, obesity, DM, rheumatoid arthritis, steroid use, depression, and previous surgery [23]. Following a thorough clinical evaluation and microbiological tests and evaluation of serial preoperative and postoperative radiographs is important. Ultrasound and CT imaging may have an additional role in image-guided fluid aspiration or tissue sampling [18, 23]. Further investigation by scintigraphy (three-phase or white blood cell [WBC] scintigraphy), FDG-PET, and MRI may be required [18].

## Imaging findings and diagnostic approach in musculoskeletal infections by direct inoculation

As a rule of thumb, radiographs, preferably in at least two orthogonal planes, are the first line of imaging modality in musculoskeletal infections [4, 9, 10, 14, 24, 31, 44, 45]. Detailed descriptions of imaging findings of musculoskeletal infectious lesions are summarized in Table 1.

### Soft tissue infections

Unless a retained infective nidus (i.e., wood, tooth fragments) is suspected, simple and superficial FB or bite/scratch wound infections rarely require imaging. Radiographs may depict soft tissue swelling, obliteration, and displacement of fat planes, and if present, findings of bone and joint infections [6, 10, 14, 24, 33, 46]. About 80% of all FB and about 98% of all radiopaque FB can be detected by radiographs (Fig. 1) [47, 48]. Although limited by size and depth within the tissue, ultrasound, with high-frequency probes (> 7.5 MHz), may show FB as hyperechoic structures with or without posterior acoustic shadowing or reverberation artifacts depending on their structure and angle of insonation. Soft tissue edema, fibrosis, granulation tissue, or abscess surrounding them may appear hypoechoic with a hypervascular rim on power Doppler ultrasound [14, 49–53]. CT is the best tool for detection of soft tissue gas, plastic, glass, and stone FB. Gas may also be detected by radiographs, ultrasound, or MRI [14, 54]. MRI serves best in the differential diagnosis and assessment of the extent of wound infections and soft tissue abscess [9, 14]. On MRI, retained FB appear as low-signal intensity structures with or without susceptibility artifacts (Fig. 2) especially on gradient echo recalled images, without any distinct anatomical shape [14, 55]. MRI has 97% sensitivity and 77% specificity for soft tissue abscesses [7]. Other accompanying imaging findings of soft tissue infections (Table 1) following FB, bite/scratch wound infections can be identical to infections from other causes [2, 6, 7, 32]. “Penumbra sign” signifies hyperintense rim compared to hypointense central cavity of an abscess on precontrast T1W images (Fig. 12) [56].

Of note, tissue vitality is best appreciated on postcontrast MR images with or without subtraction techniques. If intravenous contrast cannot be used, DWI can also help to identify devitalized tissues with the lack of tissue signal from the involved region [6, 38, 39]. Moreover, central diffusion restriction in a well-demarcated soft tissue mass on DWI indicates an abscess (Fig. 12) [6, 10, 39, 57].

### Bone and joint infections

Imaging findings of bone and joint infections after direct inoculation of microorganisms to the affected site are indistinguishable from infections that spread from contiguous infected soft tissues [4, 5, 58]. In osteomyelitis, irrespective of the route of infection, imaging findings are based on the clinical course of the disease [4, 11, 59]. Thus, patient history, demographics, and time course of the infection are particularly important. Following animal bites, radiographic findings may be subtle within the first 2 weeks, until enough bone demineralization occurs [31]. As the most robust technique with highest sensitivity and specificity, MRI demonstrates abnormal bone marrow signal on fluid-sensitive sequences near a soft tissue lesion of infection (i.e., ulcer, cellulitis, sinus tract) (Figs. 2,3, 4, and 5) [5, 6]. Ghost sign could help diagnose acute osteomyelitis or acute exacerbations of chronic disease [38, 39]. MRI can also display manifestations of chronic osteomyelitis like cloaca, involucrum, sequestrum, or sclerosis. Brodie’s abscess representing intramedullary abscess is the classic example of subacute osteomyelitis which presents as a relatively low-grade infection. However, it can also be superimposed on chronic osteomyelitis [11]. Expert Committee White Paper points out that the term “chronic osteomyelitis” should be reserved for cases with patchy areas of active infection vs fibrosis in the marrow particularly when cortical remodeling, Brodie’s abscess, sequestrum, or sinus tracts are present (Fig. 13) [6].

### Prosthetic joint, implant, and orthopedic hardware infections

Along with serial blood tests and cultures, radiographs are recommended as part of standard workup for suspected PJI. Serial radiographs have 14% sensitivity and 70% specificity; however, 50% of radiographs yield false negative results, or they may demonstrate non-specific findings such as soft tissue swelling, periprosthetic lucency, and component loosening. If present signs of gas (Fig. 11) and immature periostitis increase specificity [18], image-guided soft tissue biopsy or fluid aspiration is recommended if there are positive radiographic findings [18]. Total white blood cell count of > 3000 cells/μl and differential count of 70% neutrophils in joint aspirate are highly suggestive of PJI [18]. Positive CT findings include joint capsule and bursal distensions and periarticular soft tissue collections. Although aggressive appearing, ill-defined periprosthetic lucencies can be seen on CT, these are not diagnostic for PJI [18]. Depending on the availability, cost, radiation exposure, and operator experience, nuclear medicine examinations or MRI could be used in advanced imaging of PJI. Three-phase bone scintigraphy is the most common nuclear medicine technique. Negative results for all three phases (perfusion, blood pool, and late phase) showing the lack of osteoblastic activity or a negative WBC scintigraphy rules out PJI; however, positive bone scans have moderate sensitivity, low specificity, and low diagnostic accuracy especially within the first 5 years or in posttraumatic patients [18]. Among nuclear medicine techniques, with an overall diagnostic accuracy of > 90%, recent guidelines recommend the use of combined in vitro labelled leucocyte/bone marrow scintigraphy with standardized acquisition and imaging protocols, as the imaging modality of choice in PJI. MRI is a medium-cost, radiation-free, and widely available modality, thus a promising tool for patients who require repeated imaging [18]. However, special metal artifact reduction techniques which are commercially available by the three main MRI vendors may be required for interpretation. Positive MRI findings include soft tissue inflammatory changes, fluid collections, reactive lymphadenopathy, bone marrow edema, and synovitis (Fig. 11) [18].

## Differential diagnosis

### Soft tissue lesions

Myonecrosis in the atraumatic setting can be observed spontaneously in patients with long-term and poorly controlled diabetes. Diabetic myonecrosis commonly involves anterior thigh muscles and may mimic pyomyositis. Furthermore, aggressive infection in a closed compartment can lead to increased tissue pressure and result in myonecrosis superimposed on pyomyositis [2]. Careful evaluation of postcontrast and fluid-sensitive MR images (Fig. 14) could aid in identifying myonecrosis.

If left untreated foreign body may become encapsulated by the surrounding inflammatory tissues resulting in a granulomatous tissue response which appears as painful soft tissue swelling (Fig. 1) [4, 14, 60]. They may appear as skin nodules, hyperkeratosis with edema, mimicking soft tissue infections clinically and on imaging studies. Moreover, retained foreign bodies may result in delayed wound healing and chronic pain [9, 14].

Air trapped in dry wood particles in the early phase of FB injuries should not be mistaken for free tissue gas on imaging studies [29].

It may be difficult to distinguish soft tissue abscess from postoperative uninfected collections, myositis ossificans, necrotic soft tissue tumors, ganglia, and foreign body reactions [6, 61]. SIR guidelines recommend drainage of any abnormal fluid collection that raises suspicion for infection, is related to a fistula, or could explain the patient’s symptoms [62]. “Penumbra sign” could be helpful in distinguishing abscesses from neoplastic masses with an average specificity of 96% and a sensitivity of 27% [56].

### Joint disorders

Lead FB may dissolve and trigger reactive synovitis and inflammatory reaction if lodged within a joint, leading to degenerative and erosive lead-arthropathy that should not be mistaken for septic arthritis [9, 14, 63]. Lead-arthropathy characteristically shows hyperdense synovial hypertrophy on CT and hypointense outlines on MRI, known as “lead-arthrogram” [14, 63].

Neuropathic osteoarthropathy and osteomyelitis may have highly overlapping imaging findings. Moreover, osteomyelitis may superimpose on underlying NA. In both situations, bone marrow abnormality, joint effusion, and surrounding soft tissue edema on imaging are common. Conversely, bone marrow signal abnormalities without adjacent skin ulceration, sinus tract, or soft tissue inflammation findings are unlikely to represent infection (Fig. 6). Ghost sign on MRI is not expected in NA because bones are not just edematous, but destroyed [38]. Additionally, NA is predominantly an articular process manifesting as instability, with multiple regional joint subluxations, cysts, and debris, especially at the Lisfranc and Chopart joints. Osteomyelitis occurs predominantly at the metatarsal heads, toes, calcaneus, and malleoli, a distribution that is due to pressure points and friction, callus, and ulceration (Figs. 3 and 6) [27, 36, 37]. Of note, in up to 1% of patients who have persistent draining sinus tracts, squamous cell carcinoma may develop in the epithelial lining of the tract as a late complication of chronic osteomyelitis [4, 58].

Bone resorption at joints and entheses, hyperemia, and instability due to secondary hyperparathyroidism in chronic kidney disease may mimic infection.

Intraarticular crystal deposition in severe gout arthropathy can present with bony erosions, destruction, soft tissue swelling, and joint effusion mimicking septic arthritis. Presence of tophi, which are mass-like foci in or around the joint, should suggest gout [64]. Other inflammatory arthritides, including rheumatoid arthritis, reactive arthritis, and psoriatic arthritis, can also mimic septic arthritis and osteomyelitis on imaging, with joint effusion, joint space narrowing, erosions, and subchondral bone marrow edema. Inflammatory arthropathies are generally chronic processes with slow distention of the joint capsule due to synovial proliferation, whereas bacterial infection results in marked hyperemia and rapid joint distention resulting in aggressive appearing pericapsular edema and effusion [4].

Three-phase bone scans and FDG-PET can yield positive results in periprosthetic mechanical stress reactions as in the case of aseptic loosening and physiologic bone remodeling which may be misleading in patients suspected of PJI. Further investigation by advanced imaging methods, including WBC scintigraphy or contrast-enhanced MRI, is recommended in suspected PJI [18]. Of note, despite high negative predictive values (73.8–98%), sensitivity (26.3–10%) and specificity (47–98%) of PJI on MRI show high variability and currently there is no established consensus on MRI findings distinctly diagnostic for PJI [65].

### Bone lesions

The aggressive imaging findings of bone tumors, including bone destruction, periosteal reaction, fluid collections and necrosis, and soft tissue mass effect, should be distinguished from infection. Other skeletal disorders which may present with imaging findings of osteomyelitis such as sequestra, aggressive periosteal reactions, sclerosis, and cortical thickening and/or focal lucent lesions include Ewing sarcoma, leukemia, primary bone lymphoma, and Langerhans cell histiocytosis [4, 66]. Conversely, atypical infections such as tuberculosis and fungal and parasitic infections can cause focal bone destruction simulating tumors. Therefore, tissue sampling for both histologic and microbiologic analyses is recommended when planning biopsy [67]. As with soft tissue abscesses, Brodie’s abscess can also present penumbra sign which helps to distinguish it from bone tumors [56].

Furthermore, having familiarity with anticipated imaging findings related to surgical treatment of musculoskeletal infections would assist in recognizing normal posttreatment changes (Fig. 14).

## Conclusion

Musculoskeletal infection can affect different tissue planes with various depth and extent of involvement. Direct infection of musculoskeletal structures is commonly encountered in our daily clinical practice. Imaging appearances are variable depending on the degree of infiltration of the infectious process into different tissues and bony structures and bone marrow. Knowing the radiological findings can help in the early accurate diagnosis and choosing the appropriate treatment that lead to a significant decrease in the morbidity and mortality.

This article aimed to aid the radiologist in early diagnosis and categorization of the different patterns of musculoskeletal infection caused by direct traumatic or iatrogenic implantation and contiguous spread from infected soft tissues or adjacent joints.
